# Stigma experienced by people with mental illness in South America: an integrative review*

**DOI:** 10.15649/cuidarte.2014

**Published:** 2022-10-15

**Authors:** Raquel Helena Hernandez-Fernandes, Bruna Sordi-Carrara, Brenda Alice Andrade-Vidigal, Arthur Luís Barbosa-Martins, Sireesha Jennifer-Bobbili, Carla Aparecida Arena-Ventura

**Affiliations:** 1 . University of Sao Paulo at Ribeirao Preto College of Nursing, Sao Paulo, Brasil. Email: raquelhhfernandes@gmail.com Universidade de São Paulo University of Sao Paulo College of Nursing Sao Paulo Brazil raquelhhfernandes@gmail.com; 2 . University of Sao Paulo at Ribeirao Preto College of Nursing, Sao Paulo, Brasil. Email: brunasordi.c@hotmail.com Universidade de São Paulo University of Sao Paulo College of Nursing Sao Paulo Brazil brunasordi.c@hotmail.com; 3 . University of Sao Paulo at Ribeirao Preto College of Nursing, Sao Paulo, Brasil. Email: brendaalicevidigal@gmail.com Universidade de São Paulo University of Sao Paulo College of Nursing Sao Paulo Brazil brendaalicevidigal@gmail.com; 4 . University of Sao Paulo at Ribeirao Preto College of Nursing, Sao Paulo, Brasil. Email: arthurlbm@usp.br Universidade de São Paulo University of Sao Paulo College of Nursing Sao Paulo Brazil arthurlbm@usp.br; 5 . University of Toronto, Toronto, Canada. Email: sireeshabobbili@gmail.com University of Toronto University of Toronto Toronto Canada sireeshabobbili@gmail.com; 6 . University of Sao Paulo at Ribeirao Preto College of Nursing, Sao Paulo, Brasil. Email: caaventu@eerp.usp.br Universidade de São Paulo University of Sao Paulo College of Nursing Sao Paulo Brazil caaventu@eerp.usp.br

**Keywords:** Mental Illness, South America, Stigma, Trastorno Mental, América del Sur, Estigma, Transtorno Mental, América do Sul, Estigma

## Abstract

**Introduction:**

People with mental illness are highly stigmatized by populations around the world and are perceived to be a burden on society. As a result of stigma, many people with mental illness are discriminated against, which leads to limited life opportunities. Given that beliefs about mental illness can vary based on culture, religion, nationality and ethnicity, it is important to understand the different types of mental illness-related stigma experienced around the world.

**Materials and Methods:**

Whittemore and Knafl's (2005) methodology for integrative reviews was used to analyze 18 studies about lived experiences of mental illness-related stigma in South America.

**Results:**

Findings suggest that certain types of stigma in South America are based on gender and social norms, such as the social position of men and women in society. This leads to discrimination, isolation and violence from family, intimate partners, friends, society and health professionals. Employment is also limited for South Americans with mental illness. Other consequences, such a self-stigma, also impact the lives of people with mental illness in many South American contexts.

**Discussion:**

Family, friendship and social relationships, including health professionals, can involve processes that lead to the stigma experienced by people with mental illness.

**Conclusion:**

This integrative review highlights how mental illness related-stigma impacts individuals in South America.

## Resumen

**Introducción:**

Las personas con trastornos mentales están muy estigmatizadas por poblaciones de todo el mundo y son vistas como una carga para la sociedad. Muchas personas con trastornos mentales son discriminadas, lo que genera oportunidades de vida limitadas. Dado que las creencias sobre los trastornos mentales pueden variar según la cultura, la religión, la nacionalidad y la etnia, es importante comprender los diferentes tipos de estigma relacionados con el trastorno mental que se experimentan en todo el mundo.

**Materiales y Métodos:**

Se utilizó la metodología de Whittemore y Knafl (2005) para revisiones integrativas para analizar 18 estudios sobre experiencias vividas de estigma relacionado con el trastorno mental en América del Sur.

**Resultados:**

Los resultados sugieren que ciertos tipos de estigma en América del Sur. se basan en normas sociales y de género, como la posición social de hombres y mujeres en la sociedad. Esto conduce a la discriminación, el aislamiento y la violencia por parte de la familia, la pareja íntima, los amigos, la sociedad y los profesionales de la salud. El empleo también es limitado para los sudamericanos con trastornos mentales. Otras consecuencias, como el autoestigma, también afectan la vida de las personas con trastornos mentales en muchos contextos sudamericanos.

**Discusión:**

Las relaciones familiares, de amistad y sociales, incluidos los profesionales de la salud, pueden involucrar procesos que conducen al estigma vivido por las personas con trastornos mentales.

**Conclusión:**

Esta revisión integradora destaca cómo el estigma relacionado con el trastorno mental afecta a las personas en América del Sur.

## Resumo

**Introdujo:**

Pessoas com transtornos mentáis sao altamente estigmatizadas por populates em todo o mundo e sao vistas como um fardo para a sociedade. Como resultado do estigma, muitas pessoas com transtornos mentais sao discriminadas, o que leva a oportunidades de vidas limitadas. Dado que as crengas sobre o transtorno mental podem variar de acordo com a cultura, religiao, nacionalidade e etnia, é importante compreender os diferentes tipos de estigma relacionados ao transtorno mental vividos em todo o mundo.

**Materiais e Métodos:**

a metodologia de Whittemore e Knafl (2005) para revisóes integrativas foi usada para analisar 18 estudos sobre experiencias vividas de estigma relacionado ao transtorno mental na América do Sul.

**Resultados:**

os resultados sugerem que certos tipos de estigma na América do Sul sao baseados em genero e normas sociais, como a posigao social de homens e mulheres na sociedade. Isso leva a discriminado, isolamento e violencia por parte da familia, de parceiros íntimos, amigos, sociedade e profissionais de saúde. O emprego também é limitado para sul- americanos com transtornos mentais. Outras consequencias, como o autoestigma, também afetam a vida de pessoas com transtornos mentais em muitos contextos sul-americanos.

**Discussáo:**

Relagóes familiares, de amizade e sociais, incluindo profissionais de saúde, podem envolver processos que levam aos estigma vivenciado por pessoas com transtornos mentais.

**Conclusáo:**

Esta revisao integrativa destaca como o estigma relacionado ao transtorno mental afeta os indivíduos na América do Sul.

## Introduction

People with mental illness are usually considered to be a burden on society, and to be weak and undeserving of sympathy or empathy^1^^,^^2^^,^^3^. These stigmatizing beliefs and attitudes, which according to Goffman^4^ are social constructions, are used to label and ‘other' individuals with mental illness leading to their devaluation.

According to Link and Phelan^5^, the stigmatization process occurs as a result of labeling and stereotypes, which lead to social distance, the loss of status and finally, discrimination. Labeling occurs due to the recognition of differences in personality or behaviors of people affected by mental illness. Labeling leads to stereotypes, which are negative beliefs associated with mental illness. Social distance is used to segregate those with negative labels from those who do not share the same label. Discrimination is the resulting behaviour associated with negative attitudes and prejudice against the labeled individual, which can lead to a loss in status. Given the process of stigmatization, it is important to highlight the relationship between these elements as they typically manifest in contexts that are chacaracterized by differences in social, economic and political powers.

Thornicroft and colleagues^6^ state that stigma related with mental illness is a consequence of the lack of knowledge. In addition, these researchers argue that attitudes can be understood as prejudice, and behavior can be understood as discrimination.

However, there are different types of stigma. For instance, public stigma and structural stigma are distinct^7^. Public stigma occurs when people are prejudicial towards other people^7^. Structural stigma referes to rules, policies and procedures implemented at organizations and institutions that violate and restrict the rights and opportunities of people of stigmatized groups^7^^,^^8^. Therefore, the negative attitudes and behaviours of representatives who work at public institutions, such as health and criminal justice professionals, may actually be supported by existing policies and procesures in place^9^.

In addition, people from a stigmatized group can internalize public stigma or structural stigma, which leads to self-stigma^7^. Studies show that self-stigma directly influences a decrease in self esteem and cotributes to an individual's negative ideas about themself^10^.

In order to better understand and develop strategies to prevent and address stigma, it is important to learn about the lived experiences of people with mental illness in various contexts. For instance, stigma can be experienced in different ways in South Amercian countries due to Latin cultural values, which tend to be more protective and supportive than Anglo-American contexts^11^^,^^12^. As a result, this integrative review was conducted with the aim of identifying, analyzing and synthesizing stigma experienced by people with mental illness in South America, while taking into account the social and cultural context.

## Materials and Methods

This integrative review uses Whittemore and Knalf's methodology^13^ to appraise academic publications that investigate stigma experienced by people with mental illness in South America. It is important to highlight that integrative reviews consider data from different types of research, including empirical and theoretical studies, with a focus on methodology, theory and results^13^. Stigma experienced by people with mental illness in South America was summarized and analyzed to learn about the perceptions of people with mental illness regarding their daily experiences of stigma. As a result, this integrative review is important for better understanding stigma in South America and to develop culturally relevant and effective stigma reduction interventions.

### Problem identification

The guiding question for this integrative review is identified as: What is the stigma experienced by people with mental illness in South America? The question was formulated using the PICo strategy: (P: patient or problem; I: intervention; Co: context)^14^. In this integrative review, the patients (P) are people affected by mental illness; the intervention (I) is mental-illness related stigma and stigma reduction strategies; and the context (Co) is South America.

### Data search

The following eletronic health science databases were selected for this integrative review for the following reasons: Pubmed is the most relevant international health database; Web of Science and Scopus are multidisciplinary databases that index high-impact factor articles; PsycINFO is a recognized database in the field of Psychology; and Lilacs is a database of relevance in Latin America.

The search was conducted using MeSH (Medical Student Headings) and DeCS (Health Sciences Descriptors) descriptors. The specific terms used were “stigma”, “mental illness” and “mental disorder” as well as their derivations according to the search criteria of each database; and “Latin America”, “Brazil”, “French Guyana”, “Venezuela”, “Colombia”, “Suriname”, “Ecuador”, “Paraguay”, “Argentina”, “Uruguay”, “Bolivia”, “Chile”, “Peru” and “English Guyana”.

The inclusion criteria for the selected studies were articles which involved people with mental illness with lived experience of stigma; articles published in English, Spanish and Portuguese; primary studies published between 1991 and 2019; and articles that were readily available through the aforementioned databases. The exclusion criteria were articles classified as editorials, theses or dissertations and those that did not correspond directly to the theme of mental illness and related stigma.

### Data evaluation

Two independent reviewers conducted the review according to the established criteria^15^^,^^16^. In total, 545 studies were initially identified, however upon first review, 111 articles were excluded because they were found to be duplicates. The titles of the remaining 434 articles were reviewed using the inclusion criteria. In this second step, 250 articles were excluded. The abstracts of the remaining 184 articles were then reviewed using the inclusion criteria leading to the exclusion of 154 articles. As a final step, 30 studies were read in full, however 12 of did not meet the inclusion criteria and were excluded for the following reasons: the article was a review (2); study location was outside South America (2); studies that applied tools that measured stigma but was not related to perceived stigma by people with mental illness (4); and other themes (4).

As a result, 18 studies were included in the final sample. For data evaluation, methodological assessment tools were adapted from the literature^17^ to extract information from the studies. As well, the Briggs'^14^ instrument was used to analyze the rigour of the studies.

**Figure 1:**
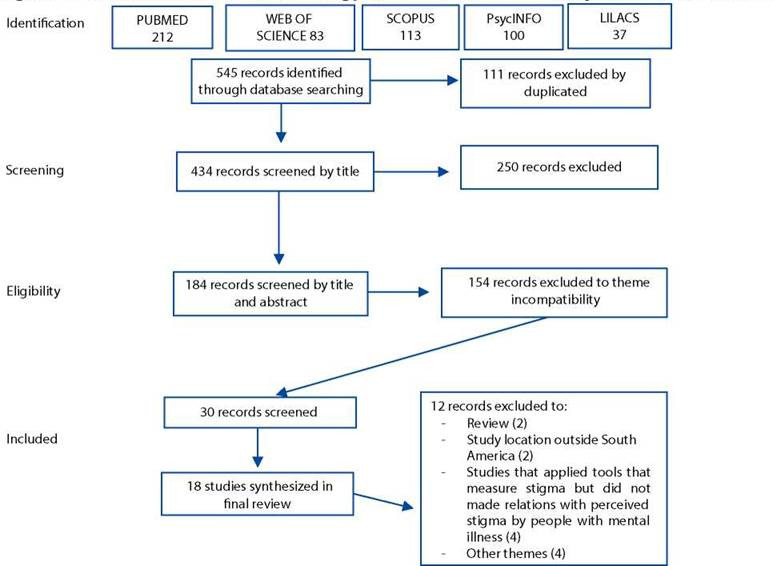
Summarizes the search strategy and the article selection phases of this review.

### Data analysis

The articles were analyzed, sorted, codified, categorized and summarized using an instrument developed by Ursi and Galvao^17^. The articles were classified according to authors, year of publication, country, sample characteristics, objectives, methods, main results and level of evidence (Table 1). The articles were then categorized according to ideas related to the interpretation of data. The Data-set was stored in Mendeley Data^18^.

## Results

Of the selected articles (n = 18), most were qualitative studies (n = 14), two were classified as quantitative studies and a few (n = 2) were classified as mixed methods (both quantitative and qualitative). In terms of location, the majority of studies were conducted in Brazil (n = 13), two studies (n = 2) were conducted in Peru, one study (n = 1) was conducted in Brazil, Argentina and Colombia, one study (n = 1) was conducted in Chile, and one study (n = 1) was conducted in Argentina, Brazil, Chile, Spain, England and Venezuela, however only the data from Argentina, Brazil, Chile and Venezuela were included in this review. Most studies involved a unique sample of people with mental illness, in particular bipolar disorder, schizophrenia and eating disorders (n = 13), followed by studies that included family members, neighbors, health professionals and caregivers in the sample (n = 5). The majority of qualitative studies used interviews as the data collection method. The mixed method studies (quantitative and qualitative) and quantitative studies used tools and questionnaires (n = 4). There was a prevalence of descriptive studies (level of evidence IV) (Table 1) that showed a low level of scientific evidence^19^ and the majority of studies were published in Brazilian Portuguese.

**Table 1:** Characteristics of selected studies

Authors/Year/Origin	Sample characteristics	Aim	Methods	Main results	Level of Evidence
Vázquez et al. (2010) Argentina, Brazil and Colombia	Two-hundred and forty-one participants with bipolar disorder were recruited from three Latin American countries (Argentina, Brazil, and Colombia)	To investigate the impact of self-rated stigma and functioning in patients with bipolar disorder in Latin-America.	Functional impairment was assessed with the Functioning Assessment Short Test (FAST) and experiences with and impact of perceived stigma was evaluated using the Inventory of Stigmatizing Experiences (ISE).	Higher scores of self-perceived stigma were correlated with lower scores of functioning. After multiple regression analysis, being on disability bene t, current mood symptoms and functioning were associated with self-perceived stigma.	VI
Robbilard (2010) Peru	General population participating in gender - specific social support groups (n=12 women/5 men); people with severe and persistent mental illness (n=22).	First, understand the gendered social cues which produce the stigma in mental illness enacted by the general population of a poor, urban district in Lima, Peru. Second, it highlights the influence of gender on the everyday experiences of those with a severe and persistent mental illness and the related stigmatization.	Combination of ethnographic and qualitative methods including a field ethnography.	In a society like that of Peru where gender roles are segregated into specific social and economic fields, gendered expectations shape both the experience of a severe and persistent mental illness and the stigmatization of people with such a mental illness in a gender-specific way.	IV
Mascayano et al. (2015) Chile	Twenty people identified as having a severe mental illness.	Explores the cultural underpinnings that create and maintainn stigmatizing attitudes towards severemental illness in Chile.	In-depth interviews developed using the ‘Scale of Perceived Discrimination and Devaluation’, and the ‘What Matters Most’ framework were conducted. Interviews were coded and discussed until agreement was reached, then analyzed by an independent reviewer to determine inter-rater reliability.	A key factor shaping stigma among women was the loss of capacity to accomplish family roles (i.e. take care of children). Or men, cultural notions of ‘Machismo’ prevented them from disclosing their psychiatric diagnosis as a means to maintain status and ability to work. A protective factor against stigma for men was their ability to guide and provide for the family, thus ful lling responsibilities attributable to ‘Familismo’. Social appearances could play either a shaping or protecting role, contingent on the social status of the individual.	IV
Volz et al. (2015) Brazil	Three people with mental illness.	Reflect on the importance of social inclusion through work in the reduction process social stigma for the illness.	Interviews and ethnography.	Social inclusion through work is the main means for the positive identification of the people with mental illness and to reduce of social stigmatization by the illness.	IV
Nunes and Torrenté (2009) Brazil	Patients with mental illness, family members and health professionals.	To analyze stigmatization processes and types of violence experienced by individuals with mental illness.	Qualitative study was carried out, based on individual interviews with users and focus groups with family members and professionals at five psychosocial care centers in the municipalities of Itaberaba, Lauro de Freitas, Salvador, Vitória da Conquista, and Aracaju, Northeastern Brazil, in 2006-2007. The analysis categories were constructed based on the stigma concept proposed by Go man, and four types of violence were systematized interpersonal, institutional, symbolic and structural.	Users and family members recounted examples of disqualification, reprimands, embarrassment, humiliation, negligence and physical aggression that had the aims of domination, exploitation and oppression. Professionals reported that people who suffer from mental disorders remain the target of prejudice that is culturally ingrained and naturalized. The main consequence is continuation of their isolation from social life as a form of “treatment” or as an excluding attitude manifested by discriminatory reactions in the form of rejection, indifference and verbal or physical aggressiveness.	V
Batista and Silva (2015) Brazil	10 users of a care network after release from psychological internment, their relatives and neighbors.	To investigate another of these moments that feed the academic debate on disease and social identity:from patient to “cause”.	Socio-an¬thropological eldwork was conducted between 2007 and 2010 with users of a care network after release from psychological internment, their relatives and neighbors.	Some villagers are considered doidos (“loonies”) without having been admitted as “patients” to the local inpatient facility. Others are “users”, registered at an outpatient service; or “clients”, when they are frequent users. Some are called bonequeiros (“trouble-makers”), “nervous”, or barulhentos (“noisy crackpots”) because of their behavior in public. Finally, by becoming the object of comments by people on the street, they also become “cases,” which are eventually discussed at the mental care facilities, thus becoming “clinical cases.” Mental disorders are as relevant to the management of stigmatized social identity as surnames and nicknames.	IV
Gasparini et al. (2013) Brazil	Five people with mental illness.	To know the meanings of mental illness for individuals with psychotic disorders, hospitalized in a general hospital.	Descriptive study with a qualitative approach. Five people formed the sample. It Was used a semi-structured interview and graphical representation in order to obtain the information. The Data was sent to content analysis, of thematic type.	The surveyed was reported to mental illness, as: a result of divine punishments and witchcraft; a reality charged with suffering and di culties, the result of losses and experiences from childhood, something di cult to explain, laden with stigma, misunderstanding, distrust and disqualification; presence of symptoms; imiting daily activities and disabling for work.	IV
Wainberg et al. (2016) Brazil	Sexually active adults attending 8 public outpatient psychiatric clinics in Rio de Janeiro (n = 641)	To examine the relationship between gender, severe mental illness diagnosis, and stigma experiences related to sexuality among people in psychiatric out patient care.	Interview. Stigma mechanisms well-established in the literature but not previously examined in relation to sexuality were measured with the Mental Illness Sex Stigma Questionnaire, a 27-item interview about stigma in sexual situations and activities.	Experiences of stigma were reported by a majority of participants for 48% of questionnaire items. Most people reported supportive attitudes toward their sexuality from providers and family members. Those with severe mental illness diagnoses showed greater stigma on individual discrimination and structural stigma mechanisms than did those with nonsevere mental illness diagnoses, whereas there was no diference on the social psychological processes (internalized stigma) mechanism. Regardless of diagnosis or gender, a majority of participants devalued themselves as sexual partners.	IV
Moreira and Melo (2008) Brazil	30 interviews with patients with mental illness in a public hospital in Fortaleza, Brazil were analyzed.	To describe part of the results of the first year of a longitudinal project about the lived experience of stigma among those with mental illness in Northeast Brazil.	Critical phenomenological method,	The presence not only of stigma but of self-stigma in the lived experience of mental illness. In addition to stigma and self-stigma, behaviors of shame, isolation and keeping the illness in secret are developed because of the pejorative image of mental illness, in the traditional comprehension of stigma as being associated to madness. Besides this comprehension, the lived experience of stigma, as well as of self-stigma appears also qualitatively related to the “invisible” character of mental illness	IV
Setti et al. (2019) Brazil	31 participants with a diagnosis of schizophrenia	To assess the effect of the Coming Out Proud (COP) intervention in individuals with the diagnosis of schizophrenia.	A pilot study of 3 2-hour group lessons (6-12 participants) per week Individuals were selected from three specialized outpatient services in São Paulo, Brazil; 46 people were willing to participate, 11 dropped out during the intervention and 4 were excluded due to low intelligence quotient (IQ), resulting in a final sample of 31 participants. Outcomes were assessed before (T0/baseline) and after (T1/directly) after the COP intervention, and at 3-week follow-up (T2/3 weeks after T1). We applied eight scales, of which four scales are analyzed in this article (Coming Out with Mental Illness Scale (COMIS), Cognitive Appraisal of Stigma as a Stressor (CogApp), Self-Stigma of Mental Illness Scale- Short Form (SSMIS) and Perceived Devaluation-Discrimination Questionnaire (PDDQ)).	People who completed the COP intervention showed a significant increase in the decision to disclose their diagnosis (22.5% in T0 vs 67.7% in T2). As to the perception of stigma as a stressor, mean values significantly increased after the intervention (T0 = 3.83, standard deviation (SD) = .92 vs T2 = 4.44, SD = 1.05; p = .006). Two results had marginal significance: self-stigma was reduced (T0 = 3.10, SD = 1.70 vs T2 = 2.73, SD = 1.87; p = .063), while perceived discrimination increased (T0 = 2.68, SD = .55 vs T2 = 2.93, SD = .75; p = .063). This study suggests that the COP group intervention facilitated participants’ disclosure decisions, and the increasing awareness of stigma as a stressor in life may have facilitated their decision to eventually disclose their disorder. The results raise questions that require further analysis, taking sociocultural factors into account, as stigma is experienced differently across cultures.	VI
Pinheiro and Spink (2004) Brazil	2 people with mental illness that use the primary health care	To explores the meaning of mental su ering for users ofmental health services, taking integrality of care as context.	To explores the meaning of mental su ering for users ofmental health services, taking integrality of care as context.	To explores the meaning of mental su ering for users ofmental health services, taking integrality of care as context.	IV
Souza and Santos (2014)Brazil	12 wonen with eating disorder.	To understand the positioning games of health care service users and how it a_ects the professio-nal-patient relationship.	The social constructionist perspective guided this research. The patients of an assistance service in eating disorders were interviewed.	The analysis of the material showed how the diagnosis works to crystallize stigmatizing versions of the patients. The concept of “relational being” was offered for health professionals relational scenarios between professional and patient that could include the concept of self as a movement and not stability.	IV
Elkingtonet al. (2010)Brazil	98 outpatients with severe mental illness.	To examine the associations between perceived mental illness stigma and HIV risk and protective behaviors among adults with severe mental illness (SMI) in Rio de Janeiro, Brazil.	Quantitative with application of tools. Measured mental illness stigma across three domains (‘‘Personal Experiences,’’ ‘‘Perceived Attractiveness,’’ and ‘‘Relationship Discrimination’’), and examined the relationship between experiences of stigma in each domain and HIV risk and protective behaviors	Those who reported greater ‘‘Relationship Discrimination’’ stigma were significantly more likely to be sexually active and to have unprotected sex; they were significantly less likely to report deliberately having fewer partners as a way to protect themselves from HIV. The role of stigma in unprotected sexual behavior should be examined further and considered in any HIV prevention intervention for people with SMI.	VI
Wagner et al. (2011)Argentina, Brazil, Chile,Spain, Englandand Venezuela.	146 people with schizophrenia (55% men) and 80 caregivers (75% women	To examine existen-tial questions in the daily life of people with schizophrenia and their caregivers.	Qualitative study with focus groups.	Four omnipresent existential themes were identified from the discussions: the need for personal development and to nd meaning in life; the need to be respected and not suffer discrimination or stigma; the conflict resulting from the loss of autonomy; the importance of understanding the illness and recognizing it as an illness. The existential questions were closely associated with objective needs, such as the lack of occupational opportunities and employment, which generally result in a life without meaning.	IV
Vedana et al. (2017)Brazil	46 Brazilian adults with mental disorders	To understand the meaning of stigma for people with mental disorders.	A qualitative study. Data were collected through semistructured interviews and nonparticipant observation and submitted for a thematic analysis with symbolic interactionism.	Stigma was considered as an experience of incomprehension and suffering. The society has difficulty in empathizing, respecting differences and understanding the extent of the suffering of people with mental disorders. Participants recommended anti-stigma strategies that included promoting knowledge and respecting differences.	IV
Onocko-Campos et al. (2013)Brazil	30 health users with mental illness and 1 health professional.	To report aspects of a qualitative study that had the opportunity to intervenein care practices in three major Brazilian cities.	Qualitative study with interviews and focus groups.	From the recorded material, the paper analyzed some situations that, among other things, attested to the difficulty of avoiding the exercise of power over users via the administration of psychotropic drugs. Little dialogue about drugs, and the existence of stigmatization spaces where user rights are inhibited or “accepted with caution,” was also detected in the services surveyed	IV
Oliveira, Facina and Júnior (2012)Brazil	10 patients with schizophrenia hospitalized in a general hospital, diagnosed with the disorder for at least ve years	To understand the reality of living with schizophrenia from the account of people who live with it.	Qualitative with interviews. The thematic analysis was used for the content analysis.	It was identified the category “Living with schizophrenia” and six themes, which concern: the knowledge of the disease, the symptoms, the hardship of living with the illness, the stigma, the family and the religion. In conclusion, this work can allows broader look at people with schizophrenia, since the knowledge about the disease and its implications occurred from the perspective of who live with such suffering on a daily basis.	IV
Cavero et al. (2018)Peru	4 psychologists, 22 primary health care providers (PHCPs) and 37 patients of antenatal care, tuberculosis, HIV/AIDS, and chronic diseases services from six primary health care centers.	To understand the offer of mental health care at the primary care level, collecting the views of psychologists, primary health care providers (PHCPs), and patients, with a focus on health services in which patients attend regularly and who present a higher prevalence of mental disorders.	A qualitative study. Semi-structured interviews were conducted with psychologists, PHCPs, and patients working in or attending the selected facilities.	A high perceived need for mental health care was noted. PHCPs acknowledged the emotional impact physical health conditions have on their patients and mentioned that referral to psychologists was reserved only for serious problems. Their approach to emotional problems was providing emotional support (includes listening, talk about their patients’ feelings, provide advice). PHCPs identified system-level barriers about the specialized mental health care, including a shortage of psychologists and an overwhelming demand, which results in brief consultations and lack in continuity of care. Psychologists focus their work on individual consultations; however, consultations were brief, did not follow a standardized model of care, and most patients attend only once. Psychologists also mentioned the lack of collaborative work among other healthcare providers. Despite these limitations, interviewed patients declared that they were willing to seek specialized care if advised and considered the psychologist's care provided as helpful; however, they recognized the stigmatization related to seeking mental health care.	IV

Source: Adapted table from Ursi's instrument^17^

The studies were grouped into two categories of experienced stigma: stigma involving family members, society and health professionals; and reflections regarding experienced stigma. 

## Discussion

### Stigma involving family members, society and health professionals

Some studies included in the review demonstrated that people with mental illness experience various behaviours linked to stigma, such as discrimination^20^, including violence, by individuals that are close to them, such as friends, neighbors and family^21^, as well as health professfionals^22^^)^ and society in general^23^.

Rejection, abandonment and discrimination by family members were the most commonly reported stigmas by people with mental illness^23^^,^^24^ which make their lives difficult^24^. In addition, one article highlighted the restrictions of rights placed on people with mental illness by their family members, such as attending school. According to a study by Vedana and colleagues^25^, people with mental illness reported that they feel they are perceived to be foolish, irresponsable and incapable by close friends and family. As a result, people with mental illness often reported that there is progressive isolation from their circle of friends, leading to exclusion and rejection^22^^,^^24^^,^^25^. The study by Nunes and Torrenté^21^ shows that in addition to rejection, people with mental illness reported feelings of indifference directed towards them by their neighbors.

Structural stigma was found in health care organizations and among health professionals, including discrimination in health services, maltreatment, neglect, refusals of care, and in extreme situations, death. For example, in a study by Onocko-Campos and colleagues^27^, some participants with mental illness reported that they were threatened with hospitalization if they did not accept taking medication. This study also reported several problems associated with unequal power relations between people with mental illness and health professionals, such as coercion, fear, and the use of inaccessible technical language regarding mental illness and treatment.

Also in relation to health professionals, stigma is experienced when people with mental illness with psychiatric and psychological symptoms seek help from health services^22^. A study by Pinheiro and Spink^28^ corroborates these findings by stating that people with mental illness are often discredited and their symptoms are attributed to organic and physical causes. In the study by Onocko-Campos and colleagues^27^, people with mental illness shared that health professionals, particularly doctors, selectively listen to patient complaints and are only concerned with organic aspects. According to a study by Souza and Santos^29^ about people with eating disorders, psychiatric symptoms have less value to health professionals than organic symptoms. In addition, participants in this study reported seeing situations in which psychiatric symptoms and consequent behaviors were seen by health professionals as ways of manipulation. The study pointed out that when organic causes for pain were not found, doctors labeled causes to be psychological and made assumptions that the person made a choice to suffer. The study by Wagner and colleagues^24^ also aligns with this perspective, as participants with schizophrenia reported that their personality traits were often associated with their symptoms by health professionals. As well, the lack of attention paid by health professionals to the physical symptoms of mental illness does not allow space for symptoms other than psychotic ones, leading to the repression of people with schizophrenia.

Studies also indicated that people with mental illness are often discredited. Participants in the study by Vedana and associates^26^ reported that health professionals were often doubtful about the existence of a mental illness and claimed that “agitation” and “laziness” were the causes of their patient's psychiatric symptoms. Participants also reported that they felt judged and that they had chosen to have a mental illness and also had complete control over their own recovery. In addition, six participants in the study by Cavero and colleagues^30^ shared that they feel misunderstood by psychologists working at public mental health services, who tend to be impatient and deal with people with mental illness very quickly and without adequate communication. The study by Onocko-Campos and colleagues^27^ suggests that some mental health and primary care professionals perceive people with mental illness to be incapable of understanding information about medications and treatment, which is a violation of their rights.

In terms of stigma communicated by society in general, people with mental illness feel misunderstood and discredited because of their conditions^31^^,^^32^.

An interesting aspect that emerged in the study by Vedana and colleagues^26^ is that people with mental illness felt stigma more strongly when those close to them distanced themselves and labeled them, giving excessive value to the flaws or limitations that they presented to the detriment of their mental illness, without considering progress and achievements.

Another form of stigma is the labelling of individuals with mental illness. A few Brazilian studies included in this review found that stigmatizing terms, such as “crazy”, were used to identify people with mental illness^33^^,^^31^, or those who cause confusion or are aggressive and violent^33^. The study by Vedana and colleagues^26^ indicate that people with mental illness experience stigma by others through verbal expressions, looks, laughter, and derogatory nicknames that communicate rejection, disrespect and contempt.

An interesting finding from this review indicates that mental illness related stigma may also be associated with gender. One study carried out in Peru^21^ suggests that people who are stigmatized due to their mental illness may also be stigmatized due to their failure to fulfill traditional gender roles. For example, if men are unemployed due to their mental illness, their manhood is questioned and they are perceived to be useless and criminal (“vagabond”), because they are unable to fulfil their role as a provider for their family. In the case of women with a mental illness, many are no longer welcomed in groups that she was once a part of and tend to be regarded as irresponsible and unable to care for her own children^21^^,^^34^.

Another study carried out in Chile by Mascayano and colleagues^34^ found that cultural factors, such as social and gender norms, are strongly associated with mental illness related stigma. For example, since mental illness may make it impossible for some men to work, they may feel stigmatized by their family members and their community for being unable to fulfil traditional male roles of being strong and a provider. This study indicates that the capacity to work, financial stability and mental illness are associated since employment has great social value. Therefore, people who do not work due to conditions related to mental illness experience stigma because they are considered to be unsuccessful by family members and the community, and are dismissed by employers who may believe that maintaining or hiring a person with a mental illness can compromise their business' social prestige^34^. The Brazilian study by Wagner and associates^24^ corroborates these findings by indicating that people with schizophrenia consider it very difficult to find a job due to stigma. Comparing with another culture, these results are similar to those found in the study by Brouwers and associates^35^, which shows that Chinese employers are more likely to not employ people with mental illness than employers in the United States and Hong Kong. This situation is probably attributed to Chinese cultural being more conservative and less open to talk about mental illness^36^.

Another form of stigma experienced by those affected by mental illness is stigma associated with a diagnosis. A study by Volz and colleagues^37^ indicate that people may be reduced to their illness or diagnosis, which eventually leads to social exclusion. Vedana and colleagues^26^^)^ also suggest that stigma associated with certain diagnoses may also be extended to the use of psychotropic drugs and psychiatric treatments. Another Brazilian study focused on patients with eating disorders showed that those with nervous bulimia perceive that their diagnosis is commonly associated with negative personality traits, leading to stigmatization^29^.

The study by Volz and associates^37^ argue that people affected by mental illness can suffer discrimination at institutions that offer mental health treatment services. According to Volz and associates^37^, family members and the community in general may label people with a mental illness as “crazy”, however they are often thought to become “even crazier” by accessing services at health care institutions. In the study by Cavero and colleagues^30^, participants with mental illness reported that some people think that psychologists are only for “crazy people”, which is indicated by how people speak about mental health treatment services in Peru.

Findings also suggest that religion may promote stigma associated with mental illness. For instance, one participant in a Brazilian study believed that his mental illness was linked to demonic forces, which represents a divine belief in punishment, which is also the belief of the social group to which he belongs^31^. The study by Oliveira, Facina and Júnior^25^ also emphasizes that mental illness is typically attributed to spiritual causes. Comparing with other cultures, similar results were found in the study by Chaudhry and Chen^38^, in which it was evidenced that South Asian religious traditions attribute mental illness to divine causes. This can hinder treatment since many conservative religions do not support the use of drug treatment services because they believe that only prayer can help people with a mental illness to recover.

Another form of stigma perceived by people with mental illness is in relation to romantic relationships and sexuality. The study by Wainberg and colleagues^20^ indicated that both women and men with a severe mental ilness people experience discrimination and structural stigma when they attempt to talk about their sexual lives with health professionals. In addition, study participants stated that regardless of whether they have a mild or severe mental illness, they are never regarded as romantic or sexual partners in a relationship. This study corroborates findings presented by Elkington and associates^23^, which indicate that some participants also experience increased stigma in relation to sexual attractiveness.

Wagner and colleagues^24^ found that participants understand the presence of structural stigma, such as inadequate government investments in health services, as well as the low availability of specialized health professionals.

Finally, another type of mental illness related stigma is associated with autonomy. The study by Wagner and colleagues^24^ presents speeches from patients with schizophrenia who indicate that the lack of autonomy enforced by health professionals (for example, by prohibiting them from doing simple, daily tasks) reinforces the belief that they are incapable.

### Reflections regarding experienced stigma

The articles included in this integrative review highlight the lived experiences and life consequences of stigma experienced by people with mental illness from their perspective. As a result, self-stigma is identified as a detrimental impact of stigma communicated by family, friends, health professionals and society^25^.

A study by Vázquez and associates^39^, carried out in Argentina, Brazil and Colombia, indicate that the stigma experienced by people with bipolar disorder has a direct impact on personal actions and daily functioning, suggesting there is a vicious cycle between the two. For instance, the more stigma perceived by people with bipolar disorder, the greater the impact on daily actions and functioning, such as productivity at work, dealing with finances and interpersonal relationships, cognitive functioning, and acting in an autonomous manner. The study also indicates there is a significant relationship between perceived stigma and the prevalence of depressive and hypomanic symptoms.

In terms of autonomy, the study by Wagner and colleagues^24^ shows that people with schizophrenia who suffer stigma feel incapable and unmotivated to make their own decisions. Thus, this reduced ability to manage their own lives leads many patients with schizophrenia to be unable to identify their physical and mental issues or manage their own treatment, which can contribute to their prolonged impairment.

An inability to work is another detrimental impact associated with experiencing stigma. Gasparini and associates^31^ state that people with mental illness feel they are perceived as being incapable of maintaining employment. In addition, the study by Moreira and Melo^32^ indicate that people with mental illness believe they are worthless and powerless, causing them to feel imprisoned within concrete and symbolic limits imposed by societal expectations associated with mental illness.

Another consequence of mental illness related stigma is the denial of a diagnosis. According to the study by Volz and associates^37^, those with a mental illness diagnosis tend to conceal their illness so others do not know that they are suffering. This is mainly due to the fear of stigma that can result after people discover their mental illness^37^. In another study, by Moreira and Melo^32^, some participants indicated they do not share that they have a mental illness with others because they do not want to be treated as a “crazy” person. Studies by Vedana and colleagues^26^ and Setti and associates^40^ suggest that denying the diagnosis is a form of protection that people with a mental illness use in order to be socially accepted. By avoiding the disclosure of a diagnosis, controlling symptoms, as well as adopting socially accepted behaviors, people with a mental illness are able to avoid the impacts of stigmatization^26^.

In the interesting study by Gasparini and colleagues^31^, one research participant did not accept that she had a mental illness, because that would mean she would have to accept the associated stigma and detrimental impacts. Since the participant associates mental illness with “madness” and those who are incapable, she is, in fact, reinforcing stigma. Souza and Santos^29^ corroborate this finding by showing that the social burden of a diagnosis causes people with mental illness to be perceived like the mental illness itself, often causing the denial of the diagnosis, similar to the participant in the study by Gasparini and colleagues^31^.

Another finding by Gasparini and colleagues^31^ indicates that self-stigma can lead to isolation.

In the study by Vedana and colleagues^26^, the authors suggest that people with a mental illness internalize stigma by feeling strange, “crazy”, useless, inferior, different and abnormal. In addition, some participants reported that they feel ashamed of themselves, which is a typical scenario that leads to isolation. This study corroborates findings from Moreira and Melo^32^, which shows that research participants felt ashamed for living with a mental illness, which prevents them from establishing relationships with others, leading to isolation.

Another consequence of stigma experienced by people with a mental illness is the low self esteem they feel in relation to their perceived low sexual attractiveness. The study by Wainberg and associates^20^ shows that more than half of the participants believed that their mental illness had a negative impact on their opportunities for sexual relationships. A little less than half of the participants also reported that they felt less attractive than others because of their mental illness. The study by Elkington and colleagues^23^ confirms these results, stating that people with severe mental illness feel less sexually and physically attractive, thereby restricting opportunities for romantic relationships. Participants in this study also reported that they perceive stigma about attractiveness to relationships. In this sense, they feel that they are not attractive to people that they want to have romantic relationships.

In terms of romantic relationships, Elkington and colleagues^23^ state that self-stigma is related to risky sexual behavior. In this study, mental illness related self-stigma is associated with a greater likelihood of engaging in unprotected sexual activities and behaviors, increasing the risk of acquiring and transmitting HIV. In addition, this study also suggests that people who experience greater stigma and discrimination are more likely to engage in sexual activity to obtain or maintain a relationship, and are less likely to negotiate condom use.

According to the study by Pinheiro and Spink^28^, the limited access to health services is another reflection of the stigma experienced by individuals affected by mental illness. Study findings revealed that health professionals at several health services in Brazil do not attempt to understand the suffering reported by service users, limiting true access to certain health services.

An important aspect appears in the study of Souza and Santos^29^ that brings a participant with nervous bulimia who shared some experiences, demonstrating the results of stigma by health professionals. In this sense, the patient reports that when health professionals say that it only depends on her overcoming the mental illness, she feels not worthy of living, because she feels that she cannot deal with her own pain.

Finally, the study by Wagner and colleagues^24^ found that the anxiety in research participants with schizophrenia is largely due to the stigma that this illness carries.

**Figure 2:**
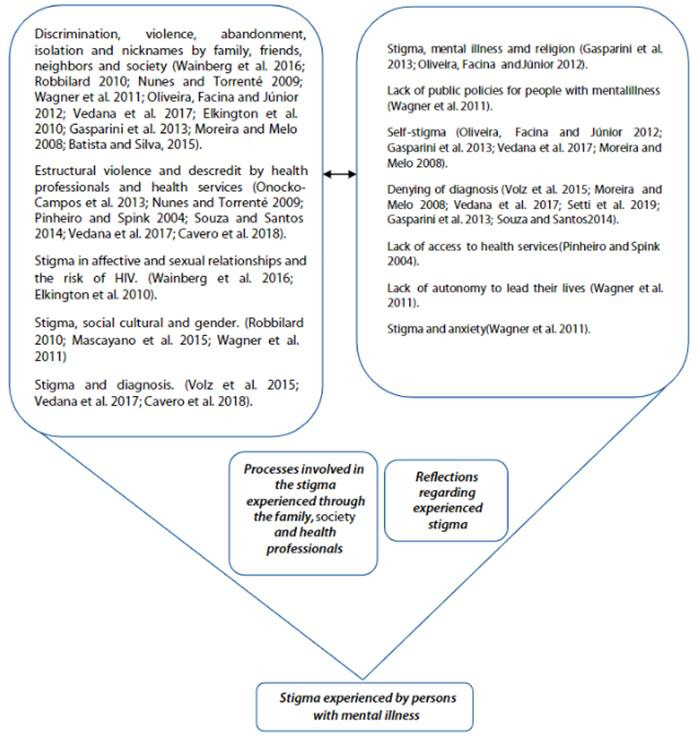
Shows the synthesis of categories found in this review.

## Conclusion

This integrative review draws attention to the impacts of stigma on the lived experiences of individuals affected by mental illness in South America. Based on the findings, stigma may lead to the abandonment of people with mental illness by family members and the communities. On the other hand, family members and health professionals tend to act in a paternalistic manner when interacting with people with a mental illness, what can also lead to violation of their human rights. In different situations, health professionals may not provide space for persons with mental illness to share their feelings. In addition, society has a history of isolating and discriminating them, what also decreases their chances of recovery. As a result, many feel isolated, discriminated against, and are perceived to be unemployable, what can result in their inability to lead their own lives.

Findings also show that certain cultural, social and gender norms influence mental illness related stigmatization in South American countries. According to traditional gender norms, a man who does not work due to a mental illness is considered weak while cultural norms suggest that mental illnesses may be due to demonic forces. Women usually are seen as unable to take care of children. Religion also appeared as a form of experienced stigma, since mental illness is understood as a divine or demonic cause. Romantic relationships and sexuality have proven to be fields where stigma is also present, so that people with mental illness are not seen as potential romantic or sexual partners.

Another interesting form of reported stigma was inadequate government investment in health services and in specialized health professionals. In sum, findings also suggest that stigmatizing experiences can reflect and greatly affect the lives of people with mental illness, leading to self-stigma, self-isolation, low self-esteem, disbelief, and denial of a diagnosis, which prevents individuals from seeking care from health services.

All of these results found in this review demonstrate how stigma is experienced in South America, bringing experiences from social, family, health services and personal contexts. This review was restricted to the search in five databases. Therefore, an inclusion of other databases and of a greater number of countries and regions may be important for the development of future studies on the theme.
